# Effect of Moderate Electric Fields on the Physical and Chemical Characteristics of Cheese Emulsions

**DOI:** 10.3390/gels9090747

**Published:** 2023-09-14

**Authors:** Ipek Altay, Lucas Sales Queiroz, Naaman F. Nogueira Silva, Aberham Hailu Feyissa, Federico Casanova, Jens J. Sloth, Mohammad Amin Mohammadifar

**Affiliations:** 1 Research Group for Food Production Engineering, National Food Institute, Technical University of Denmark, Søltofts Plads, 2800 Kongens Lyngby, Denmark; 2 Centro de Ciências da Natureza, Universidade Federal de Sao Carlos (UFSCar), Buri 18245-000, São Paulo, Brazil; 3 Research Group for Analytical Food Chemistry, National Food Institute, Technical University of Denmark, Søltofts Plads, 2800 Kongens Lyngby, Denmark

**Keywords:** ohmic heating, experimental design, Cheddar cheese, green processing

## Abstract

Cheese powder is a multifunctional ingredient that is produced by spray drying a hot cheese emulsion called cheese feed. Feed stability is achieved by manipulating calcium equilibrium using emulsifying salts. However, the increased demand for ‘green’ products created a need for alternative production methods. Therefore, this study investigated the impact of ohmic heating (OH) on Cheddar cheese, mineral balance, and the resulting cheese feed characteristics compared with a conventional method. A full factorial design was implemented to determine the optimal OH parameters for calcium solubilization. Electric field exposure and temperature had a positive correlation with mineral solubilization, where temperature had the greatest impact. Structural differences in pre-treated cheeses (TC) were analyzed using thermorheological and microscopic techniques. Obtained feeds were analyzed for particle size, stability, and viscosity. OH-treatment caused a weaker cheese structure, indicating the potential removal of calcium phosphate complexes. Lower component retention of OH_TC was attributed to the electroporation effect of OH treatment. Microscopic images revealed structural changes, with OH_TC displaying a more porous structure. Depending on the pre-treatment method, component recovery, viscosity, particle size distribution, and colloidal stability of the obtained feeds showed differences. Our findings show the potential of OH in mineral solubilization; however, further improvements are needed for industrial application.

## 1. Introduction

Cheese powder is commonly used in various food products like biscuits, sauces, soups, and bakery products, either as a flavoring agent or as a functional ingredient. In the manufacturing process, a hot cheese emulsion known as ‘cheese feed’ is produced as the spray dryer feed. This feed consists of minced cheese, water, and various ingredients such as emulsifying salts (ES). The addition of calcium-chelating agents like ES ensures the uniformity and colloidal stability of cheese feed, which is crucial for the final quality of the powder [1]. By sequestering calcium from the aqueous phase, ES reduces the concentration of free calcium ions in the cheese feed. Consequently, the calcium equilibrium is disrupted, leading to the solubilization of calcium from the paracasein matrix. As a result, the caseins show enhanced hydration and an increase in hydrodynamic volume [2]. However, the growing trend of clean-label food products reduces the desirability of utilizing ES in cheese powder production.

Due to the increasing demand for the use of ‘green’ processing techniques, novel technologies such as ohmic heating (OH), which involves a moderate electric field (MEF) process, have become popular within the food and bioprocessing industries [3]. OH involves passing an alternating electric current through a food material having non-zero electrical conductivity, where electric energy is converted into thermal energy within the sample. Thus, OH can be regarded as a technology for generating internal thermal energy, rather than solely relying on heat transfer via a medium [4]. The primary factors that affect OH processing are the electric field strength as well as the electrical conductivity of the sample, which is influenced by factors such as temperature, viscosity, and ionic dissociation. Temperature is a particularly important factor during OH due to its impact on ion mobility [5].

Furthermore, OH processing ensures uniform and rapid heating, typically occurring over a span of seconds to minutes, providing a notable advantage over conventional heating methods [6]. This feature provides a significant advantage over conventional heating methods. Other benefits of the OH process include—but are not limited to—the controllable heating rate, energy efficiency, the non-thermal effects of the electric field, adjustable design opportunities, and greater nutrient retention [5,7]. Due to its advantages over conventional methods, the technology of OH has been increasingly used for various purposes in the food industry, including pasteurization, sterilization, and pre-treatment for improved extraction yields [4,8]. 

Several studies have investigated the application of OH in dairy-based products such as dairy beverages [9], sweet whey [10], acid milk gels [11], and in the production of Minas Frescal cheese [12]. However, there is a lack of research on the effect of OH treatment on cheese samples and their mineral balance. 

Although several studies have demonstrated the effect of the pulsed electric field (PEF) on calcium transport or dissolution [13,14,15], only a limited number of studies have suggested the possible influence of OH treatment on calcium solubilization or diffusion [11,16]. Therefore, the objective of this study was to investigate the effect of moderate electric field treatment on the structure and mineral balance of cheddar cheese and to evaluate its impact on the physical and chemical properties of the resulting cheese feed. In order to determine the optimal OH parameters for calcium solubilization, we have implemented a full factorial experimental design. Through the investigation of the potential of OH treatment to facilitate calcium solubilization, the viability of utilizing OH pre-treatment for cheese emerges as a promising avenue towards eliminating or reducing the need for ES during cheese powder manufacturing.

## 2. Results and Discussion

### 2.1. Optimization of Ohmic Heating Treatment

The temperature during the OH treatment was recorded using a data logger to calculate the temperature come-up time and the duration of exposure to the electric field (Table 4). According to the results, the heating rate was inversely proportional to the applied voltage during the OH process, with a Pearson correlation coefficient of −0.89. Therefore, the exposure to the electric field was longer for the treatments conducted with a lower voltage gradient (10 V/cm). The Pearson correlation coefficient between the voltage gradient and the electric field exposure time was calculated to be −0.84, indicating a strong negative relationship between those variables. 

The amount of Ca and P minerals in the permeate obtained after Vivaspin membrane filtration is referred to as ‘solubilized Ca and solubilized P’. According to Table 1, the solubilization of both Ca and P was significantly influenced by the voltage gradient, holding temperature, and holding time. The solubilized Ca content was not affected by the parameter interactions, whereas the solubilized P content was influenced by the interaction between holding temperature and time. 

**Figure 1:**
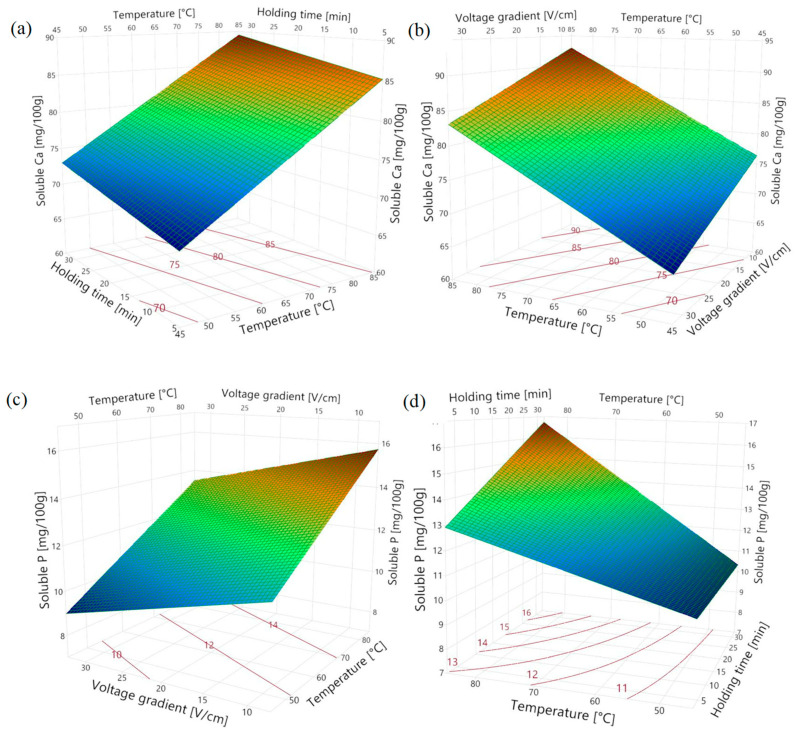
Surface plots of solubilized Ca as a function of (**a**) holding time and temperature for 20 V/cm, (**b**) voltage gradient and temperature for holding time of 17.5 min, and solubilized P as a function of (**c**) voltage gradient and temperature for holding time of 17.5 min, and (**d**) temperature and holding time for 20 V/cm.

The regression model for the solubilized Ca content showed no lack of fit, indicating a good representation of the data. However, a lack of fit was observed for the model representing the solubilized P, suggesting that there is room for improvement in predicting its behavior. This can be attributed to the non-linear relationship observed between the solubilization of P and the interaction coefficient of holding temperature and time, where at higher temperatures, the effect of holding time was more pronounced (Figure 1d). One possible approach to improve the model fit of solubilized P is to incorporate quadratic effects in a response surface model. Nevertheless, both models demonstrate a satisfactory representation of the data, identifying the significant factors that affect the solubilization of Ca and P minerals, as indicated by the R^2^_adj_ values (Table 1). 

According to Figure 1a,b, the concentration of solubilized Ca increased with the increased temperature and holding time (a positive correlation), while an increased voltage gradient resulted in a lower Ca solubilization during the OH treatment. The concentration of solubilized P also had a similar trend of the relationship with the investigated OH parameters (Figure 1c,d). It can be concluded that temperature had the most significant impact on the solubilization of both Ca and P, followed by the voltage gradient (Table 1). It is known that the electrical conductivity of foods increases with temperature due to lower viscosity, which promotes the movement of ions [7]. This could be one of the reasons that a higher amount of minerals was solubilized at elevated temperatures. The negative correlation between the voltage gradient and mineral solubilization implies a positive effect of electric field exposure on the solubilization process. This relationship arises because a decrease in the voltage gradient corresponds to an increase in electric field exposure time, which has the potential to enhance mineral solubilization [13]. The Pearson correlation coefficient for the duration of electric field exposure and solubilized Ca was 0.68 and the value for solubilized P was 0.62. Therefore, it can be inferred that the application of an electric field positively influences the release of Ca and P from the cheese structure. 

Based on the findings from the full factorial design, the best conditions determined for conducting further OH are as follows: a voltage gradient of 10 V/cm, a temperature of 80 °C, and a holding time of 30 min.

### 2.2. Analyses of Treated Cheeses

#### 2.2.1. Component Yield

**Figure 2:**
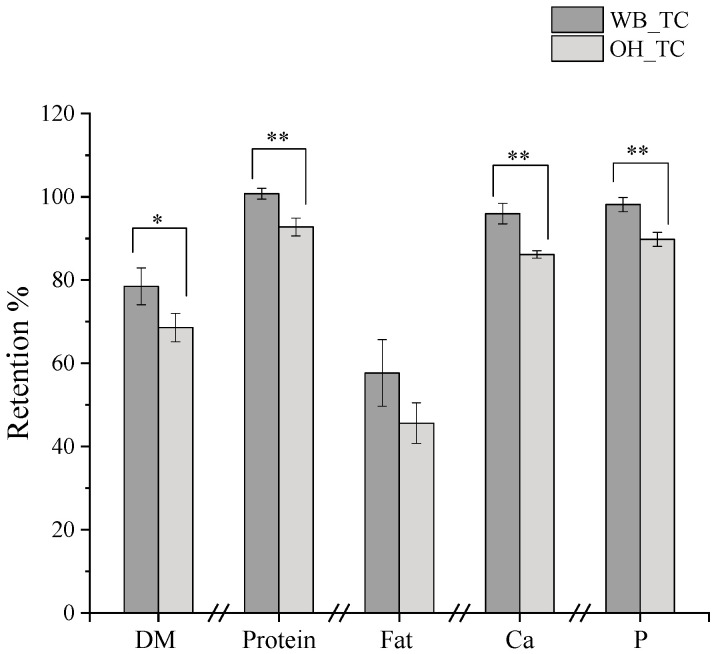
Compound retention in treated cheeses (TC) prepared with OH or WB. (*) indicates statistical significance by *p*-value < 0.05 and (**) indicates statistical significance by p-value < 0.01.

The component yield (i.e., DM, fat, protein, Ca, and P) of the OH- and WB-treated cheeses after the separation from the liquid fraction is illustrated in Figure 2. The values represent the fraction of cheese components that remained undissolved during the pre-treatment step. 

The retention of components in treated cheese was influenced by the different pre-treatment methods employed. According to our results, OH treatment resulted in an increased extraction of compounds from the cheese into the surrounding medium (DI water). In the absence of an electric field (WB_TC), the dissolution of protein was nearly negligible, whereas approximately 7% of the total cheese protein was dissolved when OH treatment was applied. Although the exact form of the extracted calcium is unclear, OH treatment (207.3 mg Ca reduction) led to around three times more reduction in calcium content of the treated cheese compared to the control treatment (60.7 mg Ca reduction). Moreover, the results presented in Section 2.2.3. indicated that the structure of OH_TC was weaker than the WB_TC, suggesting that some of the colloidal calcium phosphate (CCP) have been solubilized with the application of an electric field as structural strength is associated with CCP content [17,18]. 

It is evident that OH treatment led to the dissolution of other compounds (i.e., protein and fat) along with calcium and phosphorus (Figure 2). The efficient extraction of compounds can be attributed to the electroporation effect, which increases the permeabilization of the protein matrix due to the application of a moderate electric field [3,4]. Therefore, the difference between the final concentration of compounds for OH_TC and WB_TC was insignificant due to the higher component extraction in OH_TC. This situation provided the advantage of a similar compound composition during the cheese feed preparation, where the same amount of treated cheese was used (Table S1). 

#### 2.2.2. Microstructure of Treated Cheeses

**Figure 3:**
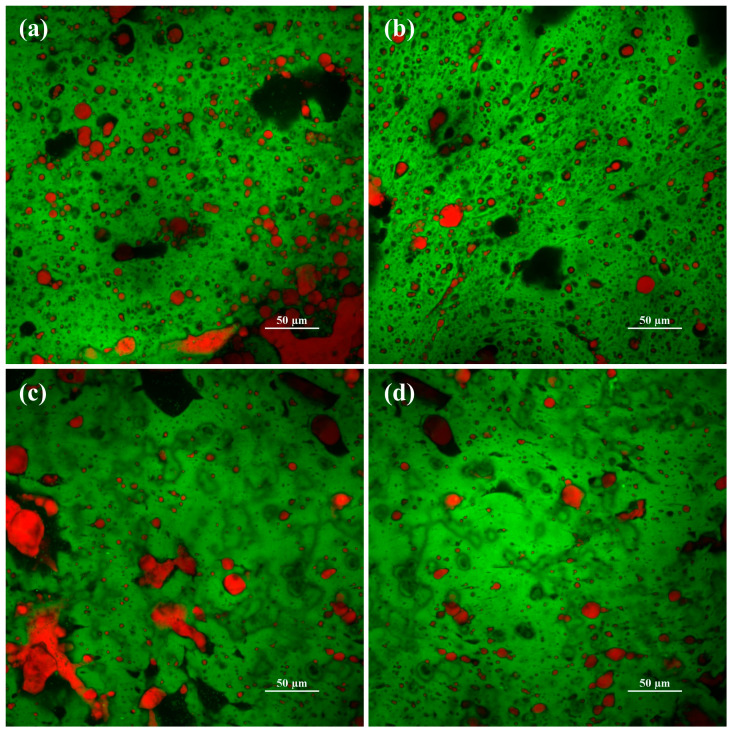
Confocal laser scanning microscopy images of OH_TC (**a**,**b**) and WB_TC (**c**,**d**) samples. Green and red colors in CLSM images indicate protein and fat, respectively. The black regions represent void areas.

Figure 3 illustrates the CLSM images of the OH- and WB-treated cheeses after storage at 4 °C overnight. Different pre-treatments led to distinct structural characteristics in the treated cheeses. The OH treatment seemed to result in a porous structure (Figure 3a,b), where fat clusters appeared to be trapped within the cheese matrix. On the other hand, the WB-treated sample (WB_TC) exhibited a protein network having fewer pores with larger fat clusters, which seemed to be loosely bound within the protein matrix (Figure 3c,d). The denser porous structure obtained via OH treatment supports the electroporation effect induced by the electric field, which increases the compound extraction from the protein matrix [6,19]. 

#### 2.2.3. Dynamic Small-Amplitude Oscillatory Rheometry

**Figure 4:**
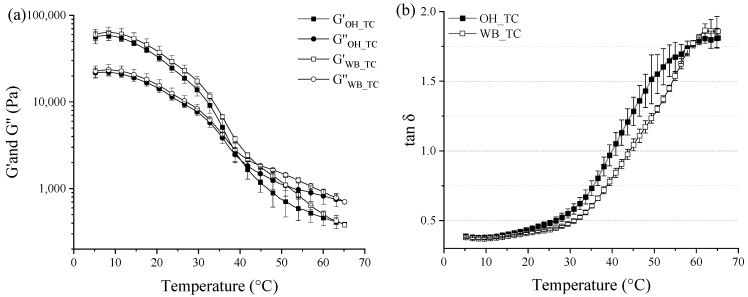
Viscoelastic moduli (**a**), and tan δ values (**b**) of cheeses treated with ohmic heating (OH_TC) or water bath (WB_TC) on heating from 5 °C to 65 °C.

Thermo-rheological analyses of the OH- and WB-treated cheeses are shown in Figure 4. According to the findings presented in Figure 4a, both the elastic (G′) and loss (G″) moduli values of the OH- or WB-treated cheeses showed a decrease as the temperature was increased. This can be attributed to the weakening of the cheese structure with the increased temperature [20]. The tan δ value of 1 represents the temperature at which the transition from a gel-like to a more fluid-like behavior occurs, known as the gel–sol transition or the G′-G″ cross-over [21]. Throughout the heating process, the OH_TC sample exhibited higher tanδ values compared to the WB_TC sample (Figure 4b), indicating a more solid-like (elastic) behavior for the WB_TC sample [22]. In addition, the OH-treated sample showed a cross-over at a temperature of 40 ± 1 °C, while the WB-treated sample had a cross-over temperature of 44 ± 1 °C (Figure 4a). Therefore, the energy required for melting the OH_TC was less than that required for WB_TC.

It is important to note that the concentration of CCP in cheese has been linked to the viscoelastic moduli, where a higher amount of CCP means a more elastic and firmer matrix [18,23]. The differences observed in the viscoelastic moduli of the treated cheeses became more distinct at higher temperatures. Likewise, previous studies reported that the effect of CCP concentration on the cheese structure becomes more pronounced at elevated temperatures [24]. These findings, together with the structural differences observed in CLSM images (Figure 3), provide evidence that the OH treatment induces alterations in the cheese structure, potentially leading to an enhanced release of calcium from the cheese matrix due to the combined effect of the electric field and high heat exposure.

### 2.3. Analyses of Cheese Emulsions

Two different types of cheese feeds were prepared using the OH- and WB-treated cheeses to evaluate the effect of the pre-treatment method on feed characteristics. 

#### 2.3.1. Yield and Composition

**Figure 5:**
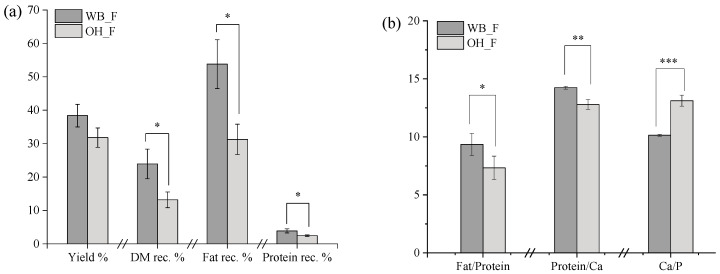
Yield and component recovery (**a**), and some component ratios (**b**) of cheese feeds prepared with different pre-treatments. (*), (**), (***) indicate statistically significance by *p*-value < 0.03, <0.01, and <0.001, respectively. WB_F and OH_F correspond to feed obtained from WB_TC and OH_TC, respectively.

Figure 5a illustrates the cheese feed yield and the percentage of components recovered into the cheese feed from the treated cheese after the sieving step. There was a noticeable increase in feed yield when the WB_TC was used in the preparation of the cheese feed (WB_F). Despite the more porous and weaker structure obtained via OH pre-treatment, the use of OH_TC during the feed preparation (OH_F) resulted in significantly lower component recovery including dry matter, fat, and protein. The higher fat recovery for WB_F could be attributed to the presence of relatively loose fat clusters in the protein matrix of WB_TC, as observed in Figure 3.

According to Figure 5b, the fat-to-protein and protein-to-Ca ratios of WB_F were significantly higher than those of OH_F. Emulsion stability can be influenced by many intrinsic factors as well as their combinations including viscosity of the external phase, particle size distribution, particle charge, and emulsifier-to-oil ratio [25]. The fat-to-protein ratio plays an important role in emulsion stability as it can influence critical factors such as the droplet size, fat surface coverage, as well as viscosity of the continuous phase [26]. An increase in fat droplet size as a result of an increased fat-to-protein mass of dairy emulsions was previously reported [27]. While higher protein or particle concentration favors the viscosity of WB_F [28], the higher fat-to-protein ratio is expected to hinder its colloidal stability. On the other hand, the lower protein-to-Ca and Ca-to-P ratios of OH_F suggest an increased susceptibility of Ca release during the feed preparation due to OH pre-treatment. 

It should be noted that the amount of solubilized protein in the medium was higher when the cheese was treated with OH. Therefore, the loss of solubilized proteins during the pre-treatment may have an impact on the final solubilized protein concentration during the preparation of the cheese feed. Both types of cheese feeds had a pH of 5.1 at 40 °C.

#### 2.3.2. Colloidal Properties 

**Figure 6:**
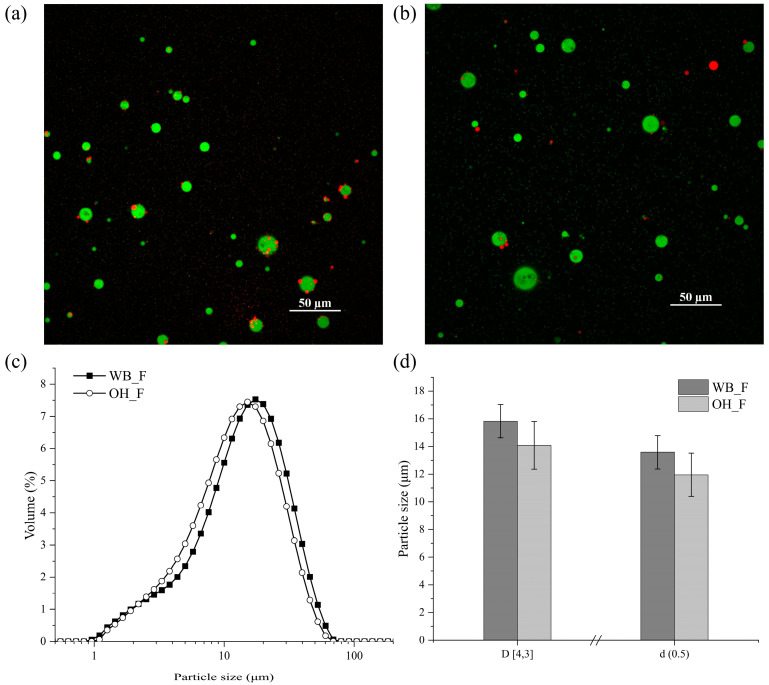
Confocal laser scanning images (CLSM) of OH_F (**a**) and WB_F (**b**), and particle size information (**c**,**d**) of feeds prepared with differently pre-treated cheeses. Green and red colors in CLSM images indicate protein and fat, respectively.

According to Figure 6c, both WB_F and OH_F exhibited similar right-skewed particle size distribution curves having one distinguishable peak, whereas the WB_F curve showed a small shoulder on the left. Additionally, both feeds had a wide range of particle sizes, indicating polydisperse emulsion characteristics. Based on our previous study on cheddar cheese feed, higher fat content was correlated with bigger particle size [29]. However, although WB_F had a higher fat content than OH_F and showed a tendency for bigger particle size, there was no statistically significant difference observed in the particle size distribution curve, d(0.5), and D [4,3] values (Figure 6c,d). As discussed in Section 2.2.1, the higher fat-to-protein ratio in WB_F could have favored a larger droplet size [27].

The CLSM images of the feeds also demonstrated similarities, as expected based on particle size information (Figure 6a,b). Notably, the OH_F sample appeared to have more fat droplets in close proximity of the proteins compared to WB_F. Previous studies on various protein types, including whey protein isolates and sodium caseinate solutions, reported significant changes in the protein structure and functionality as a result of exposure to the moderate electric field [30]. These reported changes involved alterations in the protein secondary or tertiary structure, as well as changes in the free sulfhydryl groups, hydrogen bonds, and hydrophobic interactions [30,31]. A previous study on sodium caseinate-stabilized emulsions reported that MEF-treated caseinates provided better physical stability [32]. The authors suggested that the improved functionality of emulsions was a result of the higher amount of β-structures and lower content of random coil. Moreover, the electric field induced alterations in the protein chain arrangement, and the conformational modifications were associated with the increased interfacial activity of proteins due to the exposure of hidden surface-active elements [32]. Thus, the fat-protein arrangements in cheese feed were possibly modified due to the mentioned electric field-induced alterations in the protein structure.

Both OH_F and WB_F exhibited constant viscosity throughout the analyzed shear range (Figure S1), indicating a Newtonian behavior [22]. The relatively higher viscosity of WB_F (Table 2) could be attributed to its higher dry matter content, which means a higher dispersed-phase volume fraction that leads to increased viscosity of the continuous phase [22,25].

The delta backscattering (ΔBS) value depends on the particle concentration at the region of interest [33]. Both OH_F and WB_F feeds showed an increase in ΔBS signal at the top and a simultaneous decrease at the bottom of the Turbiscan vial (Table 2). These changes indicate the occurrence of creaming phenomena [29,33], which was observed in both types of feeds over time. The peak thickness value represents the thickness of the creaming layer at the end of the measurement period [34]. In this case, WB_F exhibited a thicker creaming layer compared to OH_F, which can be attributed to its higher fat content [29]. Despite the higher viscosity of WB_F, there was no significant difference in the migration rate of the fat droplets compared to OH_F. The creaming behavior of Newtonian emulsions usually follows Stokes equation, which illustrates that droplet diameter is the most dominant factor affecting the creaming rate [25]. According to our results, it can be concluded that the viscosity of the feeds had a negligible effect on the migration rate. Since there were no significant differences in particle size between the feeds, it can be inferred that particle size played a more dominant role in determining the migration rate. Overall, it can be concluded that neither the OH_F nor WB_F feeds exhibited the desired colloidal stability.

## 3. Conclusions

The optimal OH parameters were successfully determined to enhance the solubilization of Ca and P, with higher temperatures and longer treatment times promoting solubilization while the applied voltage gradient showed an inverse relationship. The exposure to the electric field was positively correlated with the solubilization of Ca and P.

Although the exact form of released calcium was not clearly identified, OH_TC exhibiting a weaker and more porous structure compared to WB_TC suggested potentially solubilized calcium phosphate complexes (CCP). The OH treatment also led to the higher extraction of various compounds, including fat, protein, Ca, and P, with OH_TC showing a significantly lower retention of these compounds compared to WB_TC. This extraction of compounds may be attributed to the electroporation effect induced by the electric field.

During the preparation of cheese feeds, WB_F showed a higher yield compared to OH_F, while the recovery of the compounds was significantly lower with OH treatment. Despite differences in fat content, the particle size distribution of the feeds was similar. Both feeds exhibited creaming phenomena with similar migration rates, despite the higher viscosity of WB_F. This result suggests that the migration rate is primarily affected by particle size. It is worth noting that neither of the feeds prepared with OH or WB achieved the desired colloidal stability.

For the first time, OH-induced mineral and structural changes in Cheddar cheese and its effect on produced cheese feed were investigated. A further improvement for the application of OH pre-treatment could be the recycling of solubilized proteins in a liquid fraction into the feed. Methods such as centrifugation or membrane filtration could be employed for this purpose.

Overall, our study has demonstrated the potential of OH treatment to induce structural changes in cheese and has provided insights into its effects on the properties of the resulting cheese feed. These findings contribute to our understanding of the application of moderate electric fields in altering the mineral equilibria of cheese, which could eventually lead to improvements toward green processing in cheese powder production.

## 4. Materials and Methods

For all experiments, Cheddar cheese (by m/m: 39.1% moisture, 25.4% protein, 30% fat, 0.75% calcium, 0.53% phosphorus, 1.7% salt, pH 5.2, and ~3 months old) of the same batch was used.

### 4.1. Ohmic Heating Set-Up

**Figure 7:**
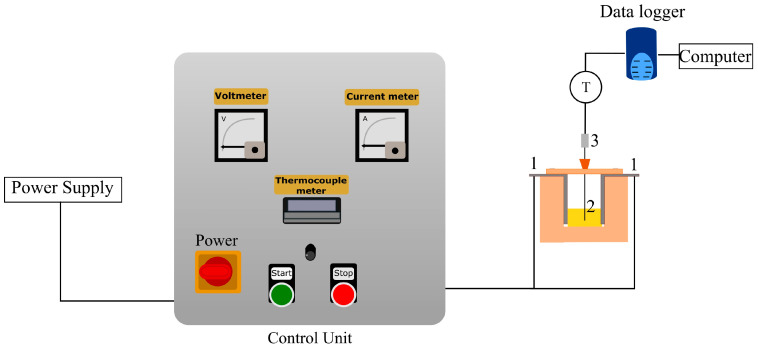
Ohmic heating system set up. 1: Titanium electrodes, 2: sample in holding unit, and 3: thermocouple.

A batch-type ohmic heater (BCH Ltd., Lancashire, UK) having a W500 grade polyethylene-polypropylene holding chamber with an adjustable distance between the titanium electrodes was used, where the chamber width was 9.5 cm (Figure 7). The OH unit had a maximum capacity to deliver 230 V and operated with an alternating current (60 Hz, sinusoidal waveform). To record the sample temperature during the treatment, a data logger (Pico, TC-08, St Neots, UK) was employed using a K-type thermocouple.

### 4.2. Determination of Ohmic Heating Parameters 

A full factorial design with a center point was employed to investigate the impact of OH parameters on the calcium and phosphorus release from cheese into the surrounding medium (deionized (DI) water) during the OH treatment. A two-level factorial design was implemented for three factors (2^3^), which were coded at two levels: −1 and +1, corresponding to the low and high level, respectively (Table 3). Additionally, a center point was included in the experimental design with a coded value of 0 (Table 4). 

The voltage gradient was calculated by dividing the applied voltage by the distance between the electrodes. Therefore, it represents the electric field strength applied to the sample in the holding chamber. The temperature factor represents the target temperature of which the sample was maintained during the period of the holding time. The temperature during the holding time was kept constant using the ‘start’—‘stop’ buttons of the control unit (Figure 7). Additionally, the electric field exposure time was calculated from the data logger curve, which represents the total duration of time that the sample was exposed to the electric field. Thereby, the effect of electric field exposure could be better interpreted.

An analysis of variance (ANOVA) and regression analysis were conducted on the model that included the main effects and interactions. The factorial equation is given in Equation (1) as:

(1) $$Y_{i}=a_{0}+\;a_{1\;}X_{1}+a_{2\;}X_{2}+a_{3\;}X_{3}+a_{12\;}X_{1}X_{2}+a_{13\;}X_{1}X_{3}+a_{23\;}X_{2}X_{3}\;\;\;\;$$

where *Y* is the response variable (i.e., solubilized Ca and P), and *X*_1_, *X*_2_, and *X*_3_ are the independent variables representing voltage gradient, temperature, and holding time, respectively. Coefficient *a*_0_ represents the intercept, the coefficients represented as *a_i_* are the main linear effects, and the *a_ij_* coefficients represent the interaction effects. ANOVA, the determination of the regression coefficients, and a lack of fit test were performed and the model’s goodness of fit was evaluated using JMP^®^ Pro 15 software (SAS Institute Inc., Cary, NC, USA). 

#### Sample Preparation for OH Parameter Determination

**Figure 8:**
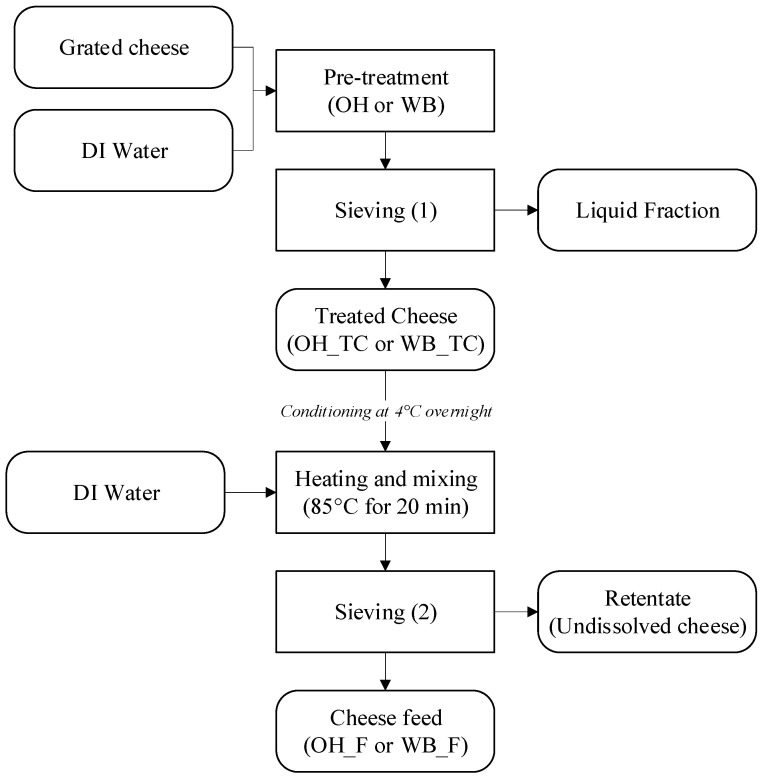
The scheme of cheese feed preparation steps. OH: ohmic heating, WB: water bath, TC: treated cheese, F: Feed.

40 g of grated cheese, conditioned at ~4 °C, was put in the holding cell, and 50 g of DI water at room temperature (~20 °C) was added in order to cover the surface of packed grated cheese in the chamber. The distance between the electrodes was set to 4 cm. After the treatment, the liquid fraction (see Figure 8) was separated from the treated cheese (TC) with a sieve and filtered using a Whatman No. 1 filter paper. To obtain the solubilized fraction of calcium (Ca) and phosphorus (P), the resulting supernatant was centrifuged at 1800× *g* for 1 h at 25 °C, utilizing the VivaSpin centrifugal concentrator equipped with a 3 kDa molecular mass cut-off membrane (Vivascience—Sartorius group, Goettingen, Germany). The obtained permeate was subsequently used for further mineral analyses (see Section 4.4.2).

### 4.3. Preparation of Cheese Feeds 

#### 4.3.1. Pre-Treatment of Cheddar Cheese 

Prior to the preparation of the feeds, the cheese was grated, conditioned at ~4 °C, and subjected to OH treatment. A conventional method–water bath (WB) treatment was used as a control treatment. After the pre-treatment step with OH or WB, the treated cheese (TC) was separated to be used in the preparation of cheese feed, as shown in Figure 8. Each treatment was conducted in triplicates. 

A batch OH unit, as described in Section 4.1, was utilized for the OH experiments. The distance between the electrodes was set to 8 cm. An amount of 200 g of grated cheese was added to the holding chamber together with 200 g of DI water, which is sufficient to cover the surface of the packed cheese. Based on the results of the full factorial design, the following parameters were applied: a voltage gradient of 10 V/cm, a treatment temperature of 80 °C, and a holding time of 30 min.

The control treatments were performed using a water bath (Julabo SW22, Seelbach, Germany). An amount of 200 g of grated cheese was put in a 500 mL Erlenmeyer flask, and 200 g of DI water at 60 °C was added to minimize the temperature come-up time and bring it closer to the OH treatment. The sample was placed in the water bath and maintained at 80 °C for a duration of 30 min.

After the pre-treatment, the obtained treated cheeses are vacuum packaged and conditioned in the fridge at 4 °C overnight.

#### 4.3.2. Cheese Feed Production 

Approximately 150 g of cheese treated either with OH or a water bath (WB) was cut into cubes measuring 1 cm × 1 cm × 1 cm. The total dry matter (DM) content of each batch was adjusted to 35% (m/m) by adding deionized (DI) water. The cheese and the DI water were mixed together for 20 min at 85 ± 1 °C using a thermoblender (HOLMs Deli Thermoblender, Svendborg, Denmark) with a blade speed of ~1000 rpm. The resulting mixture was sieved through a stainless-steel sieve with a mesh size of 500 μm (J. Engelsmann AG, Ludwigshafen, Germany—DIN ISO: 3310), where the permeate was named ‘cheese feed’. The cheese feeds were prepared in triplicates. 

### 4.4. Compositional Analyses and Component Recovery

#### 4.4.1. Proximate Composition Analyses

The total solids content of all samples was analyzed using a microwave moisture analyzer called SMART 6 ProFat (CEM Corporation, Matthews, NC, USA). The sample, which weighed approximately 2 g, was placed on a CEM glass fiber pad and heated to 105 °C until a constant weight was achieved. After determining the moisture content, the CEM pad with the sample was then analyzed for fat content using an NMR fat analyzer (ORACLE, CEM Corporation, Matthews, NC, USA). The total nitrogen content was measured using the Dumas method with a Rapid MAX N exceed^®^ analyzer (Elementar Analyse Systems GmbH, Hanau, Germany), and the crude protein content was calculated using a factor of 6.38 [35].

#### 4.4.2. Mineral Measurement

The total amounts of Ca and P present in the samples were measured using inductively coupled plasma mass spectrometry (ICP-MS) (iCAP TQ ICP-MS, Thermo Fisher Scientific, Bremen, Germany). The solid samples weighing 0.3 g and the liquid samples weighing 1 g were digested in a quartz vessel tube using a microwave-assisted digestion system (Multiwave 7000, Anton Paar, Graz, Austria) with concentrated nitric acid (SCP Science, France) [18]. Quantification was performed using the external calibration standards prepared from certified calcium and phosphorus stock solutions, with rhodium serving as the internal standard (all SCP science). Additionally, a certified reference material called DORM-5 (NRCC, Ottawa, Canada) was analyzed alongside the samples to ensure the accuracy of the analysis.

#### 4.4.3. Component and Feed Yield

After the sieving (1) step, as illustrated in Figure 8, the retention of each component (i.e., dry matter, fat, protein, Ca, and P) in treated cheeses (TC), and after the sieving (2) step, the recovery of those in the cheese feeds (CF), were calculated according to Equation (2) [29].

(2) $$\mathrm{Component}\;\mathrm{yield}\;\%=\frac{g{\;}_{\;TC\;or\;CF}\;\times \;component{\%}_{\;\;TC\;or\;CF}}{g{\;}_{cheese\;}\times \;component\%{\;}_{cheese}}$$

The yield of cheese feed production was calculated using Equation (3) [36].

(3) $$\mathrm{Yield}\%\;=\;\frac{g_{cheese\;feed}}{g_{cheese}+g_{water}}\;\times 100$$

### 4.5. Dynamic Small-Amplitude Oscillatory Rheometry

The thermorheological characteristics of the OH- and WB-treated cheeses were evaluated using a DHR-2 rheometer (TA Instruments, Hullhorst, Germany) via dynamic small-amplitude oscillatory rheometry [37]. The samples were prepared to have a diameter of 40 mm, matching the diameter of the serrated plate and the serrated platform of the instrument. Prior to the tests, the samples were conditioned in a refrigerator at approximately 4 °C. A temperature ramp was applied, starting from 5 °C and gradually increasing to 65 °C at a heating rate of 2 °C per minute. The strain value was set to 0.2%, falling within the linear viscoelastic region (LVR) of the cheese samples (data not shown). To prevent the loss of contact between the serrated plate geometry and the melting cheese, the axial force adjustment option was activated and maintained at a constant value of 1 N throughout the measurements. The results were reported as the viscous modulus (G″), elastic modulus (G′), and loss tangent (tan δ) value, which is obtained by dividing the G″ by the G′.

### 4.6. Particle Size Distribution 

The particle size distribution of the cheese feeds was assessed using a laser diffraction instrument (Mastersizer 2000, Malvern Panalytical, Malvern, UK). The refractive indices of the dispersed and continuous phases were set to 1.49 and 1.33, respectively [18]. Cheese feed drops were added to the dispersant unit filled with DI water until the final obscuration rate ranged from 10% to 12%. Particle size was determined by calculating the volume-weighed mean diameter, as shown in Equation (4).

(4) $$D\;\left[4,3\right]=\sum^{\;}n_{i}d_{i}^{4}÷\sum^{\;}n_{i}d_{i}^{3}$$

where *n_i_* is the number of particles with a diameter of *d_i_*. Additionally, the median of the distribution was reported as *d*(0.5), which represents the 50th percentile of the particle size distribution [38].

### 4.7. Colloidal Stability of Cheese Emulsions

The Turbiscan^®^ Tower (Formulaction, Toulouse, France) optical analyzer operating with Static Multiple Light Scattering (S-LMS) principles were used to evaluate the colloidal stability of cheese feeds. The instrument was equipped with an 880 nm infrared light source and two detectors that collect the transmission (T) and backscattering (BS) intensity profiles of the sample vials from bottom to top. The detectors provide the delta BS graph to identify the type of destabilization behavior, such as creaming, sedimentation, and coalescence. In addition, the migration rate was calculated as described in our previous study [29].

The colloidal stability measurements were conducted immediately after the preparation of the cheese feed. The temperature of the Turbiscan^®^ Tower was set to 60 °C, which was the temperature of the samples at the time of the measurement. The destabilization kinetics of the samples were measured every 2 min intervals for a duration of 1 h. 

### 4.8. Viscosity Measurements

The viscosity of the cheese feeds was determined using a controlled-stress rheometer equipped with a Peltier Concentric Cylinder Temperature System (DHR-2, TA Instruments, Hullhorst, Germany). The measurements were performed using a DIN concentric cylinder geometry at a temperature of 50 °C, which corresponded to the sample temperature during the measurement. The shear rate ranged from 1 s^−1^ to 200 s^−1^, and five equilibrium points were recorded for each data point.

### 4.9. Confocal Microscopy Imaging 

Confocal Laser Scanning Microscopy (CLSM) was utilized to visualize the microstructure of the cheese feed samples as well as the OH- and WB-treated cheeses. A spinning disc confocal microscope, comprising an inverted microscope (Nikon Ti2), a laser source (405/488/561/640 nm), a confocal spinning disc module (Yokogawa CSU-W1, 50 μm pinholes), a quad-band emission filter (440/521/607/700 nm), and a sCMOS camera (Photometrics Prime95B), was used to capture images of the samples. To stain the fat droplets and proteins, Nile red and FCF fast green dyes (both Sigma-Aldrich Denmark A/S, Søborg, Denmark) were added at a concentration of 0.01% and 0.001%, respectively [18]. The fat droplets were visualized using an excitation wavelength of 561 nm, with a scanning range of the emission wavelength of 607 nm. FCF fast Green was excited at 640 nm, with an emission wavelength of 700 nm.

### 4.10. Statistical Analysis

The experiments and analyses were conducted in triplicate and the results were expressed as mean ± standard deviation. To evaluate the differences between means, one-way analysis of variance (ANOVA) and Tukey’s paired comparison test were employed. A significance level of 0.05 was adopted for all analyses.

## Figures and Tables

**Table 1:** Regression coefficients and adjusted R^2^ for the reduced models.

Regression Coefficient	Solubilized Ca (mg/100 g)	Solubilized P (mg/100 g)
Constant		
a_0_	79.5115	12.364
Linear		
a_1_	−3.9058	−0.9093
a_2_	6.1942	1.6783
a_3_	1.8934	0.7843
Interactions		
a_12_	ns	ns
a_13_	ns	ns
a_23_	ns	0.5968
R^2^_adj_	0.83	0.87
RSME	3.36	0.98
*p*_model_	<0.0001	<0.0001

i = 1: voltage gradient, i = 2: holding temperature, i = 3: holding time. ns: non-significant. RSME: root mean square error.

**Table 2:** Viscosity (at 50 °C) and Turbiscan Tower (at 60 °C) results for feeds.

Parameter	WB_F	OH_F	*p*-Value
Viscosity (mPa.s)	1.5 ± 0.1 ^a^	1.1 ± 0.1 ^b^	<0.01
Migration rate (mm/h)	18.4 ± 3.7 ^a^	16.6 ± 2.9 ^a^	>0.05
Peak thickness (mm)	7.2 ± 1.3 ^a^	4.8 ± 0.7 ^b^	<0.05
ΔBS_max_ at the top (%)	14.8 ± 1.6 ^a^	22.0 ± 0.9 ^b^	<0.01
ΔBS_max_ at the bottom (%)	−29.1 ± 0.4 ^a^	−29.1 ± 0.2 ^a^	>0.05

ΔBS: delta backscattering. Values within a row with the same letter do not differ significantly (*p* < 0.05).

**Table 3:** Parameters and coded levels used in the factorial design.

Factors (Uncoded)	Factors (Coded)	Low Level (−1)	High Level (+1)
Voltage gradient (V/cm)	X_1_	10	30
Temperature (°C)	X_2_	50	80
Holding time (min)	X_3_	5	30
**Central point** *	**X_1_: 20 V/cm**	**X_2_: 65 °C**	**X_3_: 17.5 (min)**

* Added central point is reported in bold text.

**Table 4:** Full factorial design for ohmic heating, calculated come-up time, and exposure time to the electric field.

Run	X_1_	X_2_	X_3_	Come-Up Time (min)	Exposure to EF (min)
1	−1	−1	+1	2.2	4.0
2	−1	−1	−1	3.1	4.0
3	+1	−1	−1	0.3	0.5
4	0	0	0	0.7	1.2
5	−1	+1	−1	5.2	6.1
6	+1	−1	−1	0.3	0.5
7	+1	+1	−1	0.5	0.6
8	+1	+1	+1	0.2	1.7
9	+1	−1	+1	0.3	0.7
10	+1	+1	+1	0.5	1.3
11	−1	+1	−1	3.4	4.2
12	−1	+1	+1	5.0	9.3
13	+1	−1	+1	0.3	0.7
14	0	0	0	0.9	1.5
15	+1	+1	−1	0.5	0.7
16	−1	+1	+1	3.0	6.0
17	−1	−1	−1	3.1	3.7
18	−1	−1	+1	3.4	6.6

X_1_: Voltage gradient (V/cm), X_2_: Temperature (°C), X_3_: Holding time (min). Come-up time: Time it takes to reach the target temperature. Exposure to EF: Total time that the sample was exposed to the electric field.

## Data Availability

On request.

## Supplementary Materials

The following supporting information can be downloaded at: https://www.mdpi.com/article/10.3390/gels9090747/s1, Figure S1: Apparent viscosity results of feeds prepared with different pre-treated cheeses; Table S1: Component concentration of ohmic (OH) and water bath (WB)- treated cheeses (TC).
