# Supplementary material for: Effect of Moderate Electric Fields on the Physical and Chemical Characteristics of Cheese Emulsions
Source: Gels. 2023 Sep 14;9(9):747. doi: 10.3390/gels9090747 (PMC10529439; doi:10.3390/gels9090747)
Supplement: Supplementary file 1 [file gels-09-00747-s001.zip › gels-2533062-supplementary.pdf]

Supplementary Information

Table S1. Component concentration of ohmic (OH) and water bath (WB)- treated cheeses (TC).

Component	WB _TC	OH_TC
DM, %	52.0 ± 0.3	51.1 ± 0.9
Protein, %	27.9 ± 1.4	28.8 ± 1.0
Fat, %	18.8 ± 1.7	16.7 ± 1.2
Ca, mg/100g	781 ± 27	788 ± 37
P, mg/100g	571 ± 27	586 ± 35

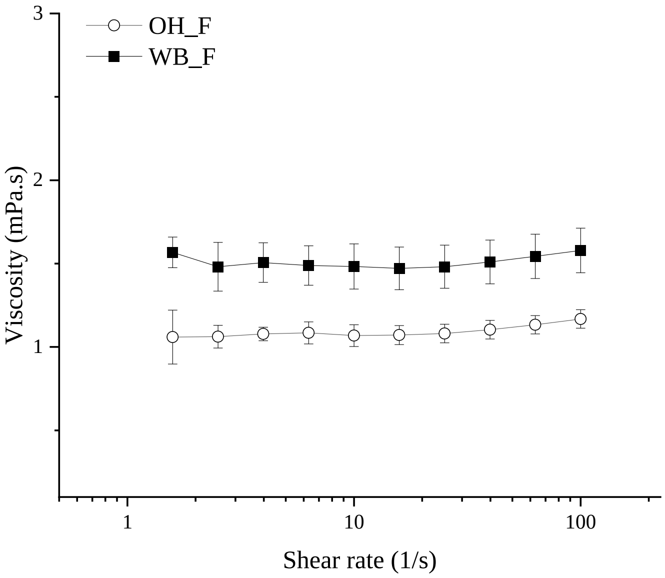

Figure S1. Apparent viscosity results of feeds prepared with different pre-treated cheeses.
